# Patient Reported Outcome Measures and Patient Reported Experience Measures for Dental Practice Quality Improvement Via Deliberative Stakeholder Consultation in the United Kingdom

**DOI:** 10.1016/j.identj.2025.103961

**Published:** 2025-10-23

**Authors:** Chiu-Yi Lin, Wendy Thompson, Lucy O’Malley, Stefan Listl, Valeska Fehrer, Matthew Byrne

**Affiliations:** a Division of Dentistry, School of Medical Sciences, Faculty of Biology, Medicine and Health, University of Manchester, Manchester, United Kingdom; b Heidelberg University, Heidelberg University Hospital, Heidelberg Institute of Global Health, Section for Oral Health, Germany

**Keywords:** Dental Care, Oral Health, Oral Care Team, Quality of Health Care, Quality Indicators, Qualitative Research

## Abstract

**Introduction and aims:**

Patient feedback have potential to drive quality improvement in dental care. However, there is a lack of clarity regarding which elements of patient feedback should be used. This study aimed to select appropriate patient-reported outcome measures (PROMs) and patient-reported experience measures (PREMs) for use in audit and feedback-based quality improvement in dental practices in the UK.

**Methods:**

A 2-phase, evidence-informed deliberative process—comprising evidence synthesis and Deliberative Stakeholder Consultations (DSCs)—was conducted. Phase 1: PROMs and PREMs candidate items were identified through systematic literature search and thematically analyzed. Phase 2: Dental patients and professionals were recruited for DSCs through purposive and snowball sampling. Inductive thematic analysis of DSC transcripts was conducted, and the final measures were agreed (5 for each dental encounter: check-up, planned treatment, and urgent care).

**Results:**

In phase 1, 672 measures (across 8 themes such as psychological/social impacts) were identified. In phase 2, 8 dentists and 5 patients participated (March-May 2024). Stakeholder priorities were as follows. Check-up appointments: dentist’s communication skills regarding treatment options and cost, and the cleanliness of facilities. Planned treatment: effective pain management during procedures and overall satisfaction with treatment. Urgent care: management of patient fear/anxiety and pain relief following a procedure. Communication of treatment progress and provision of post-operative advice were considered important in both planned and routine treatment. Waiting times for appointments were a priority across all encounters.

**Conclusion:**

Eleven measures (after removing duplicates) were selected for the encounters to offer meaningful potential for operationalizing quality improvement in dental practices. DSCs were also perceived as a useful method for prioritizing measures for quality improvement.

**Clinical Relevance:**

This study is the first to identify actionable PROMs and PREMs which can be used for audit and feedback loops in dental practices. These measures will be tested in DELIVER case studies.

## Introduction

Patient reported measures have a wide range of uses in health and technology assessment. Patient Reported Outcome Measures (PROMs) are indicators of patients' perceptions of their health conditions and related quality of life. Patient Reported Experience Measures (PREMs) are indicators of patients' perceptions of the experience of receiving healthcare.^1^ PROMs and PREMs can be used to facilitate communication between patients and health professionals for shared decision-making.^2^, ^3^, ^4^, ^5^ They can also be used^2^, ^3^, ^4^, ^5^ as outcome measures in clinical research^4^, ^5^, ^6^, ^7^, ^8^, ^9^, ^10^ or to support Quality Improvement (QI) in healthcare services.^11^ There is a growing evidence base for their use as indicators for QI in oral care services.^4^^,^^5^^,^^8^ However, determining which PROMs and PREMs are useful for QI is challenging; they must be valid, reliable,^11^ context relevant, and be able to incite an actionable change.^12^^,^^13^

When used for QI, PROMs and PREMs support audit and feedback processes. Audit assesses information about current clinical performance against an ideal standard. Feedback communicates information gathered from audit to the clinical team, with the intention of stimulating reflection and leading to a behaviour change. A 2012 systematic review of audit and feedback interventions on healthcare professional practice and outcomes indicated significant improvement.^14^ More recently, a study of feedback alone to intensive care unit teams demonstrated a 4.5% improvement.^15^

In oral health care, few coordinated, evidence-based attempts of using patient feedback for quality improvement have been made. As part of the Added Value for Oral Care (ADVOCATE) EU Horizon 2020 project, the role of patient feedback for quality improvement was developed according to an academic detailing framework; quality indicators for use in the oral care setting were developed,^4^^,^^5^ and a prototypic dashboard for implementation was tested.^12^^,^^13^ Patient reported measures included in this study focused on patient knowledge of care received, with little reference to outcomes or experiences of a dental encounter.^16^ User feedback to this dashboard suggested it was a useful tool for stimulation of discussion and reflection in practitioners, however its 46 measures were considered too expansive to be easily understood by patients and practitioners.^17^

Against this background, the DELIVER project (DELiberative ImproVemEnt of oRal care quality) aims to use deliberative processes to co-develop quality improvement solutions at practice, community and policy levels with citizens, patients, providers, and policymakers.^18^

For quality improvement in dental practices, DELIVER draws from the Clinical Performance Feedback Intervention Theory (CP-FIT). CP-FIT describes a mechanism by which feedback interventions improve performance.^16^ The CP-FIT improvement cycle comprises ten processes: goal setting, data collection and analysis, feedback, recipient interaction, perception, verification of the feedback (optional), acceptance of the feedback, intention to change behavior, behavior change (at individual and organization level) and performance improvement. Unintended consequences of the behavior change are also recognized as an additional process. Dashboards of clinical quality have been shown to be an effective tool in support of audit and feedback and that these may be useful in improving patient outcomes.^17^ Dashboards typically examine processes of care but have also been used to demonstrate patient reported outcomes, such as pain.^18^ Providing feedback through a dashboard streamlines the data collection and analysis domains of CP-FIT, can increase levels of interaction, and help practitioners to reflect on their perceptions of their performance. The information provided may stimulate an intention to change their behavior and thus deliver clinical performance improvement. In the DELIVER project, a practice dashboard is envisioned to enable dental teams to collect PROMs and PREMs from their patients, visualize their performance through quality indicators, and make plans for quality improvements following an audit and feedback logic and endorsed by an action implementation toolbox.

Drawing from a participatory research logic, the primary aim of this study was to identify, with relevant end-users, suitable PROMs and PREMs for quality improvement in dental practices in the UK for 3 types of dental encounter: routine check-up, planned treatment, and urgent care. A secondary aim of this study was to assess the quality of deliberative stakeholder consultations. This study focuses on the deliberative methods used to select the measures that will be used in a prototypic PROMs/PREMs-based quality improvement dashboard for use in general dental practices: The DELIVER Practice Dashboard (DPD).^18^ Deliberative Stakeholder Consultation (DSC) is a rapid qualitative method that may be used to address complex problems surrounding implementation of interventions in health and requires stakeholder groups work together to allow for deeper deliberations and reflections.^19^^,^^20^ In DSCs, diverse stakeholder with a range of perspectives engage in sequential group debates until broad agreement on a topic is met; this method as previously been used to develop practice-change interventions for QI in medical care and in the context of health technology assessment.^19^^,^^20^

## Methods

**Fig. 1:**
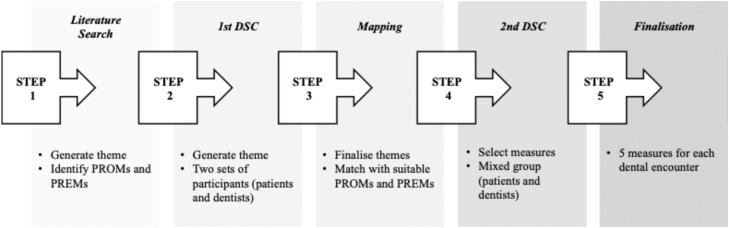
Structure of this study.

There were 5 steps in this study: literature review, DSC round 1, Mapping, DSC round 2, and finalization (Figure 1).

### Identification of measures and theme generation

PROMS and PREMs pertaining to oral care were identified previous systematic reviews of PROMs for adult dental patients^21^ and quality measures in oral and dental care.^22^^,^^23^ Further measures were identified by the DELIVER consortium through an updated systematic review of oral care quality measures (2023, unpublished: PROSPERO ID: CRD42022380315). Search terms including (“PROM” OR “PREM”) AND (“oral care” OR “dental care”), along with a snowballing technique, were used to identify PROMs and PREMs used for quality improvement in national databases that were not captured in the original search. Inclusion criteria were that the measure must: be reported by a patient; relate to an outcome or experience; and relate to oral or dental care. Additionally, the International Consortium of Health Outcome Measures (ICHOMS) Adult Oral Health Standard Set (AOHSS),^24^ having been contemporaneously ratified through a large, international consensus-forming exercise, was also included. Measure sets such as the OHIP-14^25^ were expanded to their constituent measure questions and saved in an Excel spreadsheet.

Measures were first thematically analyzed by 2 reviewers (CL and MB), resulting in initial descriptive themes. One reviewer (CL) then reviewed all measures and categorizations to refine this list. Ongoing meetings were held with the second reviewer (MB) to discuss any disagreements and finalize the themes. The finalized themes were then discussed with the wider research team to ensure consistency and relevance. These concepts formed the basis for discussions when determining which measures to select during the deliberative stakeholder consultation exercises.

### Deliberative stakeholder consultations round 1 – theme generation

The aim of the first round of deliberative stakeholder consultation was to elicit what factors were perceived as important by patients and dentists in different dental encounters. Two sets of participants (patients and dentists) were recruited through social media, advertisements, and professional networking websites. For the inclusion criteria, a patient group comprising adults who had used dental service in the last 12 months and a dentist group comprising dentists, dental healthcare professionals, registered to work in the UK across different care settings, or practice managers in the dental practices were recruited using purposive and snowball sampling. Purposive and snowball sampling were used to identify participants with in-depth understanding and experience within the specific context. This approach aligns with the purpose of the study and the chosen qualitative research method to gain rich insights into the topic. A participant information sheet was provided and participants gave written consent.

DSCs were conducted online using Microsoft Teams (moderated by MB and CL), recorded and transcribed verbatim. A topic guide (Appendix A) was constructed to guide consultations; however, participants were allowed to discuss areas of interest freely. For the patient group, the kick-off question was, “When you have visited the dentist, what do you think is important for them to know about your outcomes and experiences?.” For the dentist group, the kick off question was, “What do you want to know from your patients about their outcomes and experience?.” Three dental care encounters were identified and discussed: routine check-up appointments, planned treatment and urgent care. A questionnaire proposed by De Vries and colleagues^26^ was used to evaluate the quality of deliberations (Appendix B).

### Mapping of measures from literature to themes

For each encounter type, maps of the dominant themes and subthemes were generated. Themes generated in the first round of deliberative stakeholder consultation were cross referenced with those generated from the literature. For each theme, suitable PROMs and PREMs were shortlisted from those identified from the literature. Maps outlining the different themes arising across each encounter type were constructed to guide discussion.

### Deliberative stakeholder consultation round 2 – measure selection

A subset of the stakeholders from round 1 were recruited. An invitation was sent to them asking if they would like to participate in round 2. This comprised a mixed population either from patient or dentist groups. A mixed meeting of dentist and patient stakeholders was held to select the measures that would be used in the dashboard. The following criteria were set out:

• For each encounter type, there must be a maximum of 5 measures represented in the dashboard to ensure conciseness.
• Multiple measures may be selected within a theme, but each measure should explore a different subtheme.
• Each measure can be feasibly answered by a patient immediately after the encounter.

The meeting was conducted online using Microsoft Teams (moderated by MB and CL), recorded and transcribed verbatim. At the start of the meeting, the thematic maps were presented. The themes and subthemes in each of the encounter types were discussed and were prioritized by the group. Once 5 subthemes had been identified for each encounter, the shortlist of PROMs and PREMs for each subtheme were displayed. Where consensus could be met, i.e. all members of the group agreed to inclusion, measures were selected. After the meeting, a summary email containing the selected measures and alternatives was distributed to all members for final agreement. Transcripts of the DSCs were thematically analyzed using an inductive approach^27^ in NVivo 12, with regular meetings held by CL and MB to define and generate themes.

## Results

### Themes generated from literature

53 measure sets comprising 672 patient reported measure questions were identified (Appendix C) and thematically analyzed by 2 reviewers (CL and MB). Eight themes emerged: treatment outcome, appearance, function, pain, psychological impact, social impact, communication, and patient experience/perception of facilities

### DSC round 1: theme generation

Thirteen stakeholders engaged in the first round DSCs (7 men, 6 women; mean age 50.5 years (range: 27-69y)). The patient and dentist stakeholder groups comprised 5 and 8 participants respectively (Table 1) DSC meetings were held between March and May 2024.

**Table 1:** Characteristics of participants.

Ref.	Age	Gender	Profession
HP1	67	Male	Dentist and University clinical tutor
HP2	63	Male	Dentist and representative of Professional body
HP3	69	Male	Oral surgeon
HP4	31	Female	Dental Public Health (Wales)
HP5	65	Male	Dentist
HP6	53	Male	Academic Dentist
HP7	27	Male	Academic Dentist
HP8	55	Female	Dental Public Health (Scotland)
P1	35	Female	Patient
P2	42	Female	Patient
P3	35	Female	Patient
P4	65	Male	Patient
P5	49	Female	Patient

### Themes arising from first round deliberative stakeholder consultations

These groups were given a brief 10-minute presentation on the evidence in the literature (8 themes). Group deliberations were then initiated with pre-defined questions and were framed around specific encounter types. Five major themes were identified across all encounters and aligned to Donabedian’s domains: structure (facilities and access), process (communication and treatment) and outcome and can be seen in Table 2.

**Table 2:** Supporting quotes from DSC theme generation.

Theme	Sub-theme	Supporting Quote
Structure: Facilities	Environment	*Check-up appointment* *“I understand that, but from the patient’s point of view, the comfort of the surroundings might be an important part of their experience. That as a clinician, we should be aware of, even if we don't have the power to do very much about it.”* HP2
		*Planned treatment appointment* *“Because even now I get quite nervous when I'm going to see a dentist, I don't know why, because maybe the environmental things. So, if the environment can make me feel calm, and then, the staff, the people I'm seeing are reliable, and then it can ease some anxiety for me, I think that’s quite important.”* P2
	Location	*Check-up appointment* *“And car parking, you know, are you aware as the dentist, is there parking available, am I going to have to pay for parking to get there?”* P4
	Infection control	*Check-up appointment* *“I think I'd like* [the dentist] *to know what I think about the environment, you know, if it was clean, and hygienic, generally, if it felt like a safe environment in terms of germs, I guess.”* P4
		*Planned treatment appointment* *“The outside bit of it doesn't bother me, but if it wasn’t hygienic inside, and clean, as soon as I sat in that chair, I don't think it mattered what they did, I think I'd just subconsciously think, they’ve done everything wrong, and this isn't right.”* P1
Structure: Access	Time to appointment	*Check-up appointment* *“How easy or difficult it was to make the appointment, like, at a time that suits you. Sometimes, that’s a pain.”* P3 *“I'd want the dentist to explain to me, and say, we really need to look at this within 48 hours. Let's try and work to that.”* P1
		*Planned treatment appointment* *“And it's about, maybe, what patients think about the time between them initially contacting a practice, or initially having a check-up, between the time it would take for them to then have a procedure, or a planned appointment.”* HP4
		*Urgent care appointment* *“I suppose, before you even get to the treatment, how easy was it to access urgent dental care, and how long did you have to wait.”* HP8
	Booking system	*Check-up appointment* *“I would prefer to be able to* [book an appointment] *online at least for a check-up, which I think is generally just 15 minutes, if you could just book that yourself online, then that would be a lot easier.”* P3
Process: Communication	Current health status	*Check-up appointment* *“And I do like when I was in the clinic, or after, they show me the x-ray picture, or something visualised, so I can understand my own condition better.”* P2
	Treatment options	*Check-up appointment* *“I think, they need to explain, you know, what's required. But the technical side of it, and the options, are very important.”* P4 *“what I want to know is, have they understood what has been said to them, or what information has been given to them.”* HP4
		*Planned treatment appointment* “*Do I have a choice if I…’, ‘What's the different choice of the material?’, and ‘How does that impact the appearance?... you could have a handy leaflet, like, this is what it involves, if you want to think about, go and think about it, and if you want an appointment, I'll make it.”* P4
	Communication skills of dentists	*Check-up appointment* *“the dentist should be… interested in what I thought about his manner, his/her manner”* P4 *“Did they feel listened to during their appointment, and did they feel respected?”* HP4
	Communication skills of wider staff	*Check-up appointment* *“And possibly, the attitude of the reception staff, or whatever, they're not reception staff, or the dental staff, whatever, the reception staff.”* P4 *“And other dental care professionals that have provided care, or the receptionist, you know, other staff within the practice, that they’ve* [patients] *come into contact with, when they’ve attended for a check-up. Had they felt respected by them.*” HP4
	Time spent with patient	*Check-up appointment* *“Did you feel rushed, or did you have plenty of time? Because there are scenarios where, really, people have to have that turnover of patients, so they might feel rushed themselves, and they can't really respond to the patient wanting a lot more time.”* HP1
	Cost	*Check-up appointment* *“I think they need to explain, you know, what's required. But the technical side of it, and the options, are very important. Because, you know, you shouldn't have to be saying, well you know, you shouldn't be thinking in, you know, the NHS costs a cost, and if you begin to think, well what if I don't do this, and if it's for cost reasons… Because it's sometimes embarrassing to talk about the cost.”* P4
		*Planned treatment appointment* *“And sometimes, a filling will fall out, like a week later, something like that. And only after asking… did, I find out it was actually still covered under the NHS thing,* [for] *a certain amount of time after it had been done. And previously, I …was avoiding going, … because I didn’t realise that it was still covered up to a certain amount of time.”* P3
	Treatment progress/outline	*Planned treatment appointment* *“not just ‘Did you have what you were expecting?’, but ‘Was it explained well to you throughout the procedure?’”* HP1
		*Urgent care appointment* *“So that I left that appointment feeling like, yeah, everything happened as I expected it to, there was no sharp scratch that I wasn’t expecting, or long-lasting pain”* P1
	Risks and aftercare	*Planned treatment appointment* *if I encountered some issue, will they come back to support me, and what are the extents they can help me to fix my problem… And function-wise, and appearance-wise.”* P2 *“I think the patient’s expectation, if you take a tooth out, and they get, say, an osteitis, you know, a dry socket, then they think there's something gone wrong. It's just letting them have a means of communication.”* HP5
		*Urgent care appointment* *“I think, regarding the urgent dental care, what I really would want to feed back is, certainly [if], the after-procedure risk* [has been explained]*.”* P2 UC
Process: Treatment	Pain management	*Planned treatment appointment* *“But then there's also, was your experience of pain managed by the dental team. To have some feedback on how, you know, we explained that there might be some discomfort or pain* [during the procedure]*, but how did we manage that for you.”* HP2
	Anxiety Management	*Urgent care appointment* *“a friend of mine…had a bad experience, because her daughter is petrified of the dentist, and so the dentist got really impatient with the daughter.”* P1
Outcome	Quality of certain procedure	*Planned treatment appointment* *“Then going into detail about each specific procedure. But I suppose, it's what's important to the professional, and what's most helpful, and what, you know, would potentially improve quality for those certain procedures.”* HP4
	Social impact	*Urgent care appointment* *“I think it would be good for you to tell your dentist, if they're interested to know that what your circumstances are at that point. I've got this, but I've got, you know, I've got work here, I need to know how much time I'm going to have to take off, if I do have to take time off. I've got childcare.”* P4
	Symptom improvement	*Urgent care appointment* *“From a sort of ‘emergency pain management’-type appointment, I think the core outcome would be, has the dentist met your expectations, have they done what you came in for?”* HP7

For planned check-up appointments 3 key themes were drawn from the DSC: Communication, Facilities and Access. Communication was the dominant theme. Both groups identified the manner of dentists and practice staff, discussion of current health status, discussion of treatment options and the time spent during communication as important indicators of quality. Patients also highlighted the importance of discussions around costs. Perceptions of the facilities was another dominant theme brought up by both groups; dental professionals felt that an overall patient assessment of the comfort of the environment was important, whereas patients focused on the cleanliness and location of the practice. Regarding Access, the patient group highlighted the time taken to get an appointment and the ease of booking appointments as important markers of quality.

Five major themes were interpreted from discussions of planned treatment. Communication again predominated, however subthemes related more towards the practicalities of treatment, with adequate communication of the treatment progress (i.e. what to expect and when) and the aftercare needed for treatment deemed important. Discussions of treatment options and costs at planned treatments were also seen as important factors for some patients. Facilities were discussed by patients, with a focus on the importance of a calm and comfortable environment when undergoing planned treatment. Access, and the time to have an appointment were highlighted by the dentist group, who identified a short time between check-up and treatment appointments as a marker of quality. It was felt by both groups that the patient’s knowledge of the quality of the process of procedure would be difficult to ascertain, however the dentist group identified adequate pain management during a procedure as a suitable patient reported measure. Patient reported outcomes and perceptions were considered an important factor for planned treatment, however it was felt that the wide range of treatments on offer would each necessitate their own measure to maximize their utility. In the contexts of this dashboard, such individual measures were deemed infeasible.

Urgent care yielded a different set of priorities. Outcome was the dominant theme discussed in the context of urgent care, with subthemes exploring the social impacts of care (i.e. did the treatment allow the patients to return to a functional social state) and how well the treatment satisfied the needs of the patient. Communication was limited to adequate communication of the treatment options, alternatives, and risks. Access, and the time to get an appointment were again seen as markers of quality. Both patients and dentists, recognized that patients attending for an urgent appointment may be more likely to exhibit anxiety, and thus how well the dental team managed any anxiety was deemed important.

### DSC round 2: Final measure selection

A final DSC was held with 3 dentists and 3 patients (HP 1, HP6, HP8, P1, P4, and P5). The final measures identified are presented in Table 3.

**Table 3:** Measures selected in DSCs.

Ref	Theme (Subtheme)	Measure (Reference)	Rating Scale	Numerator	Denominator	Encounter
1	Process: Communication (Treatment options)	Did you feel sufficiently involved in decisions about your care?^28^	Yes/No	Number of survey responses where the answer is “Yes”*	Total number of survey responses.*	Check-up
2	Process: Communication (cost)	If you have to pay for your dental care, did you understand the costs of the dental care?^29^	(Yes/No)	Number of survey responses where the answer is “Yes”^	Total number of survey responses^	Check-up
3	Process: Communication (Manner of dentists)	I really felt understood by my dentist^30^	Strongly Agree/ Agree/ Uncertain/ Disagree/ Strongly Disagree	Number of survey responses where the answer is either “Strongly agree” or “Agree”^.	Total number of survey responses^	Check-up
4	Process: Communication (Treatment progress/outline)	Thinking about the procedure you have had, were you provided with sufficient information prior to the procedure that enabled you to understand what would happen?^31^	Yes/ No/ Unsure	Number of survey responses where the answer is “Yes”^	Total number of survey responses^	Planned Treatment Urgent care
5	Process: Communication (Post-operative advice)	Did you receive information, in a format that you could understand, about care after the operation?^32^	Agree/Disagree/Not sure	Number of survey responses where the answer is “Agree”^	Total number of survey responses^	Planned Treatment Urgent Care
6	Structure: Facilities (Cleanliness of facilities)	How satisfied were you with the cleanliness of the practice?^28^	Very satisfied/ Quite satisfied/ Quite unsatisfied/ Very unsatisfied	number of survey responses where the answer is either “Very satisfied” or “Quite satisfied.”^	Total number of survey responses^	Check-up
7	Overall satisfaction	The dental treatment was completed to my satisfaction.^33^	Yes/ No/ I don't know	Number of survey responses where the answer is “Yes” ^	Total number of survey responses^	Planned Treatment
8	Pain management during procedure	Was your pain managed well during the procedure?^32^	Agree/ Disagree/ Not sure	Number of survey responses where the answer is “Agree”^	Total number of survey responses^	Planned treatment
9	Pain relief after procedure	If you had toothache or a painful mouth, did you feel better after your appointment?^29^	Yes/ No/ n/a	Number of survey responses where the answer is “Yes”^	Total number of survey responses^	Urgent Care
10	Management of patient fear/anxiety	Was your anxiety managed well during the procedure?^32^	Agree/ Disagree/ Not sure	Number of survey responses where the answer is “Agree”^	Total number of survey responses^	Urgent Care
11	Waiting times for appointments	How do you feel about the length of time taken to get an appointment?^34^	It was as soon as necessary/ It should have been a bit sooner/ It should have been much sooner	Number of survey responses where the answer is “As soon as necessary^.”^*	Total number of survey responses*	Check-up, Planned treatment, Urgent Care

⁎ Numerator and denominator described in original source.

^ Numerator and denominator inferred from context of measure question.

The subthemes prioritized by the mixed group for a Routine check-up appointment were communicating treatment options; communicating cost; manner of the dentists; cleanliness of facilities and waiting times for appointments. As in the first DSC, communication dominated discussion. It was felt, by all parties, that dentists and dental care teams should ensure that they exchange clear information about their dental condition, treatment options, and costs. Furthermore, the manner of the dentist and how well the dentists were prioritized.

*“Because I think they’re more or less connected to each other, and I think it’s showing you what options are available, you know? Are you in the right practice? Are you in the safe hands of someone who really understands you?”* P5 (mixed group)

“Did you feel sufficiently involved in decisions about your care?”; “If you have to pay for your dental care, did you understand the costs of the dental care?” and “I really felt understood by my dentist” were selected from the shortlisted communication measures to satisfy these subthemes.

Whilst patients may not be familiar with all of the infection control measures employed by the practice, they perceived that the general appearance of cleanliness was a surrogate measure of how well hygiene procedures were being adhered to. “How satisfied were you with the cleanliness of the practice?” was accepted by the group as a suitable patient reported measure.

In the context of routine check-up appointments, the need for timely diagnosis to minimize treatment need was highlighted. The measure question “How do you feel about the length of time taken to get an appointment?” was selected as the most appropriate shortlisted measure. This measure was also selected in the context of planned treatment and urgent care, where timely treatment was deemed necessary to prevent worsening of oral disease.

For planned treatment, the subthemes prioritized were communication of treatment progress, communication of aftercare instructions, pain management, treatment outcome and time to appointment. Regarding communication measures for planned treatment, it was expected their dentists would address the patient's questions and concerns and describe risks and side and aftercare during a treatment appointment. For example:

*“So, still making sure we’ve got that full consent procedure going, ensuring the information has been given so you know what the risks and benefits and alternatives are.”* HP8

*“You know, was pain explained to you before your treatment and any pain management post treatment as well*.” P1 (mixed group)

“Thinking about the procedure you have had, were you provided with sufficient information prior to the procedure that enabled you to understand what would happen?” and “Did you receive information, in a format that you could understand, about care after the operation?” were chosen to satisfy these subthemes.

Effective intraoperative pain management was prioritized and highlighted as an important factor for patients to communicate with their dentists.

*“if you have pain during the procedure, you’re stuck in the chair, you’ve got to deal with it, haven’t you? I mean, it’s a good thing to* [know]*…”* P4 (mixed group)

“Was your pain managed well during the procedure?” was selected by the group.

Following previous discussions from the dentist group DSC, the mixed groups felt that having individual quality indicators for each dental procedure would be too complicated but felt that a measure of satisfaction of the outcome was important for patients to be able to express. The group agreed that the question, “The dental treatment was completed to my satisfaction” achieved this.

For urgent care, communication of treatment progress, communication of aftercare, and time to appointments were all prioritized, with previously identified measures deemed suitable for use in this context. Management of patient fear/anxiety, and relief of symptoms were also prioritized. The group felt that patients seeking urgent care may have heightened anxiety; either from a fear of the dentist themselves, or due to their presenting discomfort and anticipation of dental treatment. Anxiety management was considered a key skill, that was not necessarily held by all dentists. Measurement of this skill would aid therefore in quality improvement:

*“If they are feeling anxious, the dentist is very understanding and empathetic and understands your needs. But I think training is very crucial and I think it needs to be addressed and put in place in every dentist’s practice.”* P5 (mixed group)

“Was your anxiety managed well during the procedure?” was universally accepted.

Adequate relief of symptoms was considered by the group as the most important outcome of urgent dental care:

*“I think if it’s urgent care, I think it’s just getting you out of pain as quick as possible.”* P5 (mixed group)

The question, “If you had toothache or a painful mouth, did you feel better after your appointment?” was therefore selected.

### Quality of the deliberative stakeholder consultations

Self-report quality assessment of the deliberations was applied to ensure the quality of the deliberations. The self-report feedback showed the deliberations were of high quality, as reflected in the level of engagement and the comments from the participants. Eleven participants from the round 1 DSCs and 4 participants from round 2 DSC responded to the quality assessment survey (appendix B). The descriptive statistics indicated that participants had good engagement in the deliberations and had a high willingness to abide by the groups decision even if it differed from their own. However, it must be noted that these sessions had little impact on changing their opinion about the topic.

## Discussion

The findings of the present study provide a short and practical list of stakeholder-consented PROMs/PREMs for quality improvement in dental practices in the UK. The prioritized PROMs/PREMs slightly differed depending on the envisioned clinical encounter (routine check-up, planned treatment, and urgent care). The quality of the DSCs was found to be high, supporting their suitability for identifying quality measures.

### Comparison to previous literature

Increasing attention is being paid to quality measures for oral care in the field.^4^^,^^5^ Some research and projects have emerged on this topic. For example, stakeholders’ perspectives on quality measurement of oral health care have been explored in the literature.^35^ In particular, the EU ADVOCATE project aimed to explore quality measures for oral care to optimize the delivery of oral healthcare in EU countries.^4^ The ADVOCATE project examined the validity and reliability of selected measures for quality improvement in dental practices across EU countries.^4^^,^^5^ However, a limitation of this previous work can be seen in quality indicators which were based on a multicountry and multi-purpose consensus, resulting in a vast number of indicators which are difficult to apply in country-specific contexts of practice-based quality improvement. The extensive list of 46 measures from the ADVOCATE project may be challenging to fully implement in dental practices. In addition, a lack of dedicated patient input has been highlighted in the literature.^4^^,^^5^ The findings of this study offer a more concise and focused list of actionable measures to support the quality of dental care while enhancing feasibility and usability in real-world settings. Meanwhile, the study provides important additional insights and can help address existing knowledge gaps by incorporating perspectives from various stakeholders, including patients and dentists, with a concrete, context-specific focus on the intended use of PROMs and PREMs.

Most measures selected through the present study focus on the communication between professionals and patients. Patients from this study desired a high level of information and active involvement about their oral care, reflecting findings in the literature.^36^ Effective communication and information exchange facilitate active patient involvement and shared decision making.^37^^,^^38^^,^^39^ The majority of measures selected through the present study focuses on the communication between professionals and patients. The patients from this study desired a high level of information and active involvement about their oral care, reflecting findings in the literature.^36^ Effective communication and information exchange facilitate active patient involvement and shared decision making.^37^^,^^38^^,^^39^ Providing sufficient information on treatment options, costs, timelines, risks and benefits, not only ensures informed consent, but may lower patient anxiety, improve patient satisfaction,^40^^,^^41^^,^^42^ and improve trust.^43^

Time pressures were identified as the main barrier to providing sufficient information-communication, reflecting the literature.^44^ Given the UK context of this study, these time pressures were seen as inherent problems with NHS dentistry. Patients who exclusively used NHS services felt that it was understandable that they would have limited time with their dentist compared to those paying privately; this was reflected in concerns expressed by NHS dentists around having too little time with patients. The use of quality measures for information-communication may be therefore particularly important in time limited public health services, where information needs to be given as succinctly as possible. Whilst communication skills are taught at both undergraduate level and are recommended for continuing professional development,^45^ each dentist will have a variable level of training in this area. Tracking patient experiences and outcomes through a dashboard may allow dental teams to closely monitor their approaches to communication and institute changes to the aides and techniques they may use.

Pain management for both planned treatment and urgent care appointments, was explored within the groups in this study. It is noticeable that poor pain management could result in negative impacts on daily life and quality of life,^46^ poor health outcomes, and lower patient satisfaction.^47^ Some concerns were raised for the use of pain rating scales as quality indicators; the subjective nature of pain means it is difficult to compare between different patients, meaning in-subject changes of pain state need to be considered.^48^ Recording a change in state would require contacting patients at multiple time points and would be out of scope of the proposed dashboard. To address this and still facilitate a single point of measurement, the selected measures address how well the pain was managed. Where patients indicate that pain is not appropriately managed this would hopefully stimulate clinicians to consider pain management strategies, around effective communication, use of intraoperative pain assessment tools, and use of tailored postoperative instructions.

Waiting times for appointments was identified as a key priority for patients and dentists for each of the 3 discussed dental encounters. This may reflect the context of poor access to dental treatment in the UK that has been reported relating to the backlog of care post-COVID, and shortage of healthcare professionals.^49^^,^^50^^,^^51^ Delayed oral health assessment and treatment results in worse dental health outcomes, negative patient-provider relationships, and dissatisfaction with dental care.^52^ It is understandable that this concept was seen as a crucial indicator for patient-reported experience measures and quality improvement. Within a state funded system, this indicator may be the most challenging for a dental practice team to be able to change, given the political determinants of funding. However, given this study’s findings of patients being cognizant of the pressures of care, patients are likely to be able to pass a context-aware judgment of the reasonability of their waiting times. If this measure were to be introduced in non-state funded contexts it would be interesting to see if this measure were rated as highly as it was in the UK.

The selected measures will then be adopted into the dashboard to collect data from patients immediately after their visits. Based on CP-FIT, audit and feedback have been recognized as the foundational mechanisms for quality improvement in dental practices. By reviewing patient feedback through the dashboard, audit processes assess the quality of healthcare service delivery. The dashboard will regularly provide dental professionals with summary findings from these measures to reflect on their performance. This provides dental healthcare teams with data-driven insights into the current situation regarding the selected quality measures. This study describes a methodology to priorities existing measures of quality to ensure their contextual relevance. By directly engaging stakeholders in deep discussion and debate, the DSC method allows for collection of rich data.

### Strengths

This study represents the first results to provide a detailed and context-specific focus on the intended use of PROMs and PREMs in dental practices. The list of selected PROMs and PREMs is more concise and user-friendly compared to previous works in the literature (e.g. the ADVOCATE project). Furthermore, this study, with its highly positive evaluation of its comprehensive participatory approach that actively engaged end-users and had not been previously assessed in this field, further validates the approach used in both this study and the DELIVER project.

### Limitations

This study was carried out within the UK context, which could result in limited generalizability to other settings. To ensure transferability in other contexts, further studies in different contexts may be needed to examine the adaptation of the selected PROMs and PREMs. In the context of the DELIVER project, the identified PROMs and PREMs will also be evaluated for their applicability in the context of oral health care in Germany and adapted where needed through deliberative stakeholder consultation.

Additionally, although we aimed to include diverse stakeholders from dental practices, the sample size (n=13) and the variety of participants might be considered limited. Only dentists and individuals who are currently using or have used dental care were included in this study. Despite these, self-report quality assessment of the deliberations was used to ensure that in-depth data were collected contributing to the richness of data, which is core advantage of qualitative research. It is also important to note that these measures have been validated in the German context using the RAND instrument. Additionally, a pilot study is planned to assess their feasibility within the specific settings of the research. However, some weaknesses of deliberative stakeholder consultations have been reported in the literature, such as disproportionate reflection of the views of the most dominant voices within the group, or power dynamics causing professional’s views to dominate over patients. To detect and control for this effect, quality assessments of the deliberations were carried out after each of the DSCs, demonstrating good quality deliberations with active engagement of the participants. The use of the mixed stakeholder group allowed participants to reflect upon the views and challenges seen across the participants, to share concerns, and potential approaches or barriers to tackle these.

## Conclusion

This study identified important quality measures for different dental encounters (routine check-up, planned treatment, and urgent care) to assist dentists and dental care teams in quality improvement strategies. Measures selected through the deliberative stakeholder consultations focused on the quality of communication between dentists and patients, ease of access, and effective pain and anxiety management. Future research is warranted to describe how these measures can be adapted for other contexts. Within the DELIVER Project, the research teams will assess the feasibility of implementing these identified PROMs and PREMs in the real-world practice settings through the use of an interactive dashboard.

## Authors' contributions

CL, SL, and MB contributed to the conception and design of the work. CL and MB conducted data collection and data analysis. CL and MB contributed to interpretation of data. The authors (CL, WT, LO, SL, and MB) collectively contributed to the data interpretation. CL and MB drafted the manuscript. All authors reviewed the manuscript for important intellectual content. All authors gave final approval of the version to be published and agree to be accountable for all aspects of the work in ensuring that questions related to the accuracy or integrity of any part of the work are appropriately investigated and resolved.

## Conflict of interests

The authors declare that there is no conflict of interest.

## Funding

This work was supported by the European Union’s Horizon research and innovation programme [grant number 101057077] and the UK Research and Innovation Council under the Horizon Guarantee scheme [grant number 10048830).

## Statement of authorship

The submitting author affirms that all individuals listed as authors agree that they have met the criteria of authorship, agree to the conclusions of the study and that no individual meeting the criteria of authorship has been omitted. In order to meet the requirements of authorship, each author must have contributed to at least 1 aspect of each of the 4 criteria, as listed below. *Please note that for Criteria 1 and 2, authors only need to meet 1 of the 2 items listed*. These criteria are not to be used as a means to disqualify colleagues from authorship who otherwise meet authorship criteria by denying them the opportunity to meet criteria 2 or 3. Therefore, all individuals who meet the first criterion should have the opportunity to participate in the drafting, review, and final approval of the manuscript. Any individuals not meeting the criteria may be mentioned in the Acknowledgements section of the manuscript.

Per the criteria defined by the International Committee for Medical Journal Editors (ICJME), please note the contribution made by each author listed in the manuscript*. Please type each role into the boxes that apply. Do NOT respond with “yes or no” or “X” or your form will be returned.*

**Table:** 

Author (Last name, First Initial) e.g. Smith, J	Criteria 1		Criteria 2		Criteria 3	Criteria 4
	contributed to conception or design	contributed to acquisition, analysis, or interpretation	drafted the manuscript	critically revised the manuscript	gave final approval	Agrees to be accountable for all aspects of work ensuring integrity and accuracy
Lin, C	Contributed to conception and design	Contributed to data collection, analysis and interpretation Collectively contributed to the data interpretation	Drafted the manuscript	critically revised the manuscript	Gave final approval	Agrees to be accountable for all aspects of work ensuring integrity and accuracy
Thompson, W		Collectively contributed to the data interpretation		critically revised the manuscript	Gave final approval	Agrees to be accountable for all aspects of work ensuring integrity and accuracy
O’Malley, L		Contributed to data interpretation Collectively contributed to the data interpretation		critically revised the manuscript	Gave final approval	Agrees to be accountable for all aspects of work ensuring integrity and accuracy
Listl, S	Contributed to conception	Collectively contributed to the data interpretation		critically revised the manuscript	Gave final approval	Agrees to be accountable for all aspects of work ensuring integrity and accuracy
Fehrer, V		Collectively contributed to the data interpretation		critically revised the manuscript	Gave final approval	Agrees to be accountable for all aspects of work ensuring integrity and accuracy
Byrne, M	Contributed to conception and design	Contributed to data collection, analysis and interpretation Collectively contributed to the data interpretation	Drafted the manuscript	critically revised the manuscript	Gave final approval	Agrees to be accountable for all aspects of work ensuring integrity and accuracy

## Figures and Tables

**Table:** 

Questions	Score range, 1-10 (SD)	
	Small group	Mixed group
	(*n* = 11)	(*n* = 4)
1. Do you feel that your opinions were respected by your group?	10.0 (0.0)	9.5 (1.0)
2. Do you feel you were listened to by your facilitator?	10.0 (0.0)	10.0 (0.0)
3. Do you feel that the process that led to your group’s response was fair?	9.6 (0.9)	9.3 (1.5)
4. How willing are you to abide by the group’s final position, even if you personally have a different view?	8.7 (1.8)	9.8 (0.5)
5. How helpful did you find each of the following?		
a. Question and answer interaction with the experts?	9.5 (0.8)	9.3 (1.0)
b. The formal presentations given by the experts?	9.2 (1.1)	8.3 (1.3)
c. Discussing the issues with other participants?	9.6 (0.7)	8.3 (2.4)
6. How much did attending the session change your understanding about the topic?	7.3 (2.0)	8.0 (1.6)
7. How much did attending the session change your opinion about the topic?	6.1 (2.2)	5.8 (2.9)

**Table:** 

Abbreviation	Title	First author	Year	Total measures
OIDP	Oral Impacts on Daily Performance	Adulyanon^53^	1997	5
CEOH	Core elements of oral health	Ahonen^54^	2021	1
OES-1	one-item version of the Orofacial Esthetic Scale	Andela^55^	2022	1
GOHAI	Geriatric Oral Health Assessment Index	Atchison^56^	1990	12
	A Consensus-Based Set of Measures for Oral Health Care	Baâdoudi^8^	2017	8
Alt-CFQ	Chewing Function Questionnaire - Alternative Version	Baba^57^	2009	20
EGOHID (Part D)	European Global Oral Health Indicators Development	Bourgeois^58^	2008	5
DVSS	Dental Visit Satisfaction scale	Corah^59^	1984	10
OH-QoL	Oral Health Quality of Life Inventory	Cornell,^60^	1997	30
GR	Global Oral Health rating	Dolan^61^	2007	1
	Outcome measures to assess the effectiveness of endodontic treatment for pulpitis and apical periodontitis	Duncan^62^	2021	7
OHVS	Oral health values scale	Edwards^63^	2021	12
	Oral health care in Europe: Financing, access and provision	Winkelmann^64^	2022	2
RAND DHI	Rand Dental Health Index	Gooch^65^	1989	3
	Core outcome set for traumatic dental injuries	Kenny^66^	2018	4
PIDAQ	Psychological Impact of Dental Aesthetics Questionnaire	Klages^67^	2006	23
PDA	Patient Denture Assessment	Komagamine^68^	2014	22
SASS	Smile Aesthetics Satisfaction Scale	Lajnert^69^	2018	5
OES	Orofacial Esthetic Scale	Larsson^70^	2010	8
DIDL	Dental Impacts on Daily Living	Leao^71^	1996	36
OHIDL	Oral Health impacts on daily living	Liu^72^	2017	20
SOHSI	Subjective Oral Health Status Indicators	Locker^73^	1994	43
	Quality improvement strategies within Egyptian dental clinics	Mabrouk^74^	2019	22
OHIP-5	5 Item oral impact profile	Naik^75^	2016	5
OHQoL-UK	Oral Health Related Quality of Life-UK	McGrath^76^	2001	16
CCU-OHQoL	oral health-related quality of life in patients hospitalised in critical care	Moreno Sancho^77^	2019	3
FDI-AOHSS	ICHOM Adult oral health standard set	Ni Riordain^24^	2021	13
LORQ	Liverpool Oral Rehabilitation Questionnaire	Pace-Balzan^78^	2004	25
New-CFQ	New Chewing Function Questionnaire	Peršić^79^	2013	10
OHIP-14	Short-form oral health impact profile	Slade^80^	2006	14
OHIP-49	Oral health impact profile	Slade^81^	1994	49
DIP	Dental Impact Profile	Strauss^82^	1993	25
	Locker's Global Oral health item (Validated by Thomson)	Thomson^83^	2012	1
	Conscious sedation: guidance for health boards and dental health professionals	Welsh government^84^	2018	1
	Dental Patient experience questionnaire	Welsh Government^29^	2020	13
	Filling the Cavities: Improving the Efficiency and Equity of Canada’s Dental Care System	CD Howe Insitute^85^	2018	1
	Key health inequalities in Canada: a national portrait.	Pan-Canadian Public Health Network / Public Health Agency of Canada^86^	2018	2
	Filling the gap: a universal dental care scheme for Australia	Analysis and policy observatory^87^	2019	2
NOHSS	National Oral Health Surveillance System	Centers for Disease Control and Prevention^88^	2024	4
DQOF	Dental Quality and Outcomes Framework for 2016-17	Department of Health^28^	2016	7
	Nordic Project of Quality Indicators for Oral Health Care	National Institute for Health and Welfare (Finland)^89^	2010	2
	Clinical standard: oral surgery	NHS England^90^	2023	10
	Clinical standard for urgent dental care	NHS England^91^	2023	2
	NHSBSA practice questionnaire	NHS England^34^	2017	2
	Commissioning Dental Services: Service standards for Conscious Sedation in a primary care setting	NHS England^31^	2017	3

## Appendix A: Deliberative stakeholder consultation topic guide

1. **Introduction to the study and the research team**
  a. “Hello Mr. / Ms. Welcome to our stakeholder focus group discussion. Thank you for being here.”
  b. “My name is _ and I am a researcher at the University of Manchester. I will be conducting today’s stakeholder group discussion, and you can contact me or my colleagues if any questions arise.”
  c. “This study is to select which PROMs and PREMs will be used in the study for each of 3 encounter types (routine check-up, elective treatment, urgent care) that will be reported on in the DELIVER practice dashboard for use in primary dental care.”
  d. “I will ask you some questions concerning PROMs and PREMs in primary dental care. This is meant to be an open discussion, so please answer according to your own perceptions. Please don’t hesitate to say anything that comes to mind, even if you think it’s not relevant to us.”
  e. Information: what data will be gathered, how it will be processed, main points of the information leaflet which must be covered (e.g. voluntary participation, possibility of withdrawal of consent at any time, no repercussions if participant leaves the study before the end, data protection, rights)
  f. Address the issue of confidentiality. All information collected will be confidential and participants’ names will not be disclosed; neither will any attributions for quotes be made in a final report.
  g. Ground rules. These rules can facilitate setting boundaries for decorum (for example, mobile phones on silent) and for interaction and exchange (for example, listening to others, no interrupting, speaking up). The following are ground rules that help establish the group discussion.
    • Only 1 person talks at a time.
    • Confidentiality is assured. “What is shared in the room stays in the room.”
    • It is important for us to hear everyone’s ideas and opinions. There are no right or wrong answers to questions – just ideas, experiences and opinions, which are all valuable.
    • It is important for us to hear all sides of an issue – both the positive and the negative.
    • It is important for women’s and men’s ideas to be equally represented and respected.
  h. “I will start recording now. I will let you know when the recording is over.”
2. **A brief 10-minute presentation**
  a. *Small group deliberations:* the evidence around implementation barriers and the intervention context, with time for questions. This will also include a PROMs and PREMs list, and a maximum of 5 PROMs and PREMs for each encounter type (routine care, elective treatment, and urgent care).
  b. *Mixed group deliberations*: a summary of the deliberations in Phase 1 (including areas of disagreement and outstanding questions) will be provided by researchers/moderators.
3. **Questions**
  a. *Small group deliberations*
    • Can you introduce yourself to the group and tell us about your current role? (Icebreaker question)
    • What PREMs and PROMs have been used in primary dental care?
    • How do you feel about the selected PREMs and PROMs?
    • From your perspective, what elements of PREMs and PROMs should be included in primary dental care? Why?
    • Are there other PREMs and PROMs which would be more appropriate for quality improvement measurement in primary dental care?
    • How would these selected PREMs and PROMs facilitate or be barriers to quality improvement in primary dental care?
  b. *Mixed group deliberations*
    • Can you introduce yourself to the group and tell us about your current role? (Icebreaker question)
    • What do you think about the areas of disagreement and outstanding questions on selection of PREMs and PROMs from Phase 1?
    • How do you feel about the updated PREMs and PROMs from Phase 1?
    • Are there any other PREMs and PROMs which would be more appropriate for quality improvement measurement in primary dental care?
    • How would these selected PREMs and PROMs facilitate or be barriers to quality improvement in primary dental care?
4. **Enquiries**
  • Fact checking
  • Interpretation checking
    ○ “Did I understand it correctly that _?”
  • Information not mentioned before
5. **Closing**
  • Summarize some general themes and insight that came out of the conversation and give participants an opportunity for any final words
  • Thank them for their input
  • Inform participants when recording is over
  • Inform them how the data will be used and explain how the results will be disseminated
